# Bis{[μ-bis­(diphenyl­phosphino)methane-1:2κ^2^ *P*:*P*′]nona­carbonyl-1κ^3^ *C*,2κ^3^ *C*,3κ^3^ *C*-[tris­(4-methoxy­phen­yl)arsine-3κ*As*]-*triangulo*-triruthenium(0)} dichloro­methane solvate

**DOI:** 10.1107/S1600536809052088

**Published:** 2009-12-09

**Authors:** Omar bin Shawkataly, Imthyaz Ahmed Khan, Chin Sing Yeap, Hoong-Kun Fun

**Affiliations:** a Chemical Sciences Programme, School of Distance Education, Universiti Sains Malaysia, 11800 USM, Penang, Malaysia; b X-ray Crystallography Unit, School of Physics, Universiti Sains Malaysia, 11800 USM, Penang, Malaysia

## Abstract

The asymmetric unit of the title *triangulo*-triruthenium compound, 2[Ru_3_(C_21_H_21_AsO_3_)(C_25_H_22_P_2_)(CO)_9_]·CH_2_Cl_2_, contains one *triangulo*-triruthenium complex mol­ecule and one half-mol­ecule of the dichloro­methane solvent. The dichloro­methane solvent lies across a crystallographic inversion center leading to the mol­ecule being disordered over two positions of equal occupancy. The bis­(diphenyl­phosphino)methane ligand bridges an Ru—Ru bond and the monodentate arsine ligand bonds to the third Ru atom. Both the arsine and phosphine ligands are equatorial with respect to the Ru_3_ triangle. In addition, each Ru atom carries one equatorial and two axial terminal carbonyl ligands. The three arsine-substituted benzene rings make dihedral angles of 82.00 (6), 76.67 (7) and 66.09 (6)° with each other. The dihedral angles between the two benzene rings are 80.12 (8) and 78.34 (7)° for the two diphenyl­phosphino groups. In the crystal packing, the mol­ecules are linked together into chains down the *b* axis *via* inter­molecular C—H⋯O hydrogen bonds. An inter­molecular C—H⋯O hydrogen bond and weak inter­molecular C—H⋯π inter­actions further stabilize the crystal structure.

## Related literature

For general background to *triangulo*-triruthenium derivatives, see: Bruce *et al.* (1985 ▶, 1988*a* ▶,*b* ▶). For related structures, see: Shawkataly *et al.* (1998 ▶, 2004 ▶, 2009 ▶). For the synthesis of tris­(4-methoxy­phen­yl)arsine, see: Blicke & Cataline (1938 ▶) and for that of μ-bis­(diphenylphosphino)methanedeca­carbonyl­tri­ruthenium(0), see: Bruce *et al.* (1983 ▶). For the stability of the temperature controller used for the data collection, see: Cosier & Glazer (1986 ▶).
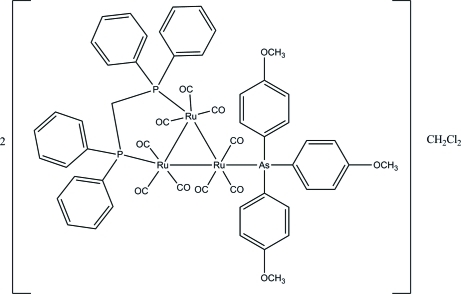


## Experimental

### 

#### Crystal data

2[Ru_3_(C_21_H_21_AsO_3_)(C_25_H_22_P_2_)(CO)_9_]·CH_2_Cl_2_
*M* *_r_* = 2756.85
Triclinic, 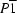

*a* = 10.7428 (1) Å
*b* = 12.6731 (1) Å
*c* = 20.6529 (2) Å
α = 95.523 (1)°
β = 101.315 (1)°
γ = 103.929 (1)°
*V* = 2645.47 (4) Å^3^
*Z* = 1
Mo *K*α radiation
μ = 1.64 mm^−1^
*T* = 100 K
0.30 × 0.23 × 0.14 mm

#### Data collection

Bruker SMART APEXII CCD area-detector diffractometer
Absorption correction: multi-scan (*SADABS*; Bruker, 2005 ▶) *T*_min_ = 0.637, *T*_max_ = 0.797
123575 measured reflections
23203 independent reflections
20247 reflections with *I* > 2σ(*I*)
*R*_int_ = 0.030

#### Refinement

*R*[*F* ^2^ > 2σ(*F* ^2^)] = 0.023
*wR*(*F* ^2^) = 0.055
*S* = 1.02
23203 reflections
679 parameters
H-atom parameters constrained
Δρ_max_ = 1.72 e Å^−3^
Δρ_min_ = −1.34 e Å^−3^

### 

Data collection: *APEX2* (Bruker, 2005 ▶); cell refinement: *SAINT* (Bruker, 2005 ▶); data reduction: *SAINT*; program(s) used to solve structure: *SHELXTL* (Sheldrick, 2008 ▶); program(s) used to refine structure: *SHELXTL*; molecular graphics: *SHELXTL*; software used to prepare material for publication: *SHELXTL* and *PLATON* (Spek, 2009 ▶).

## Supplementary Material

Crystal structure: contains datablocks global, I. DOI: 10.1107/S1600536809052088/sj2701sup1.cif

Structure factors: contains datablocks I. DOI: 10.1107/S1600536809052088/sj2701Isup2.hkl

Additional supplementary materials:  crystallographic information; 3D view; checkCIF report

## Figures and Tables

**Table 1:** Hydrogen-bond geometry (Å, °)

*D*—H⋯*A*	*D*—H	H⋯*A*	*D*⋯*A*	*D*—H⋯*A*
C53—H53*C*⋯O2^i^	0.96	2.60	3.346 (2)	135
C56—H56*A*⋯O7	0.96	2.60	3.116 (3)	114
C22—H22*A*⋯*Cg*1^ii^	0.93	2.91	3.5901 (16)	131
C53—H53*B*⋯*Cg*2^iii^	0.96	2.85	3.6951 (17)	147

Symmetry codes: (i) 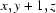; (ii) 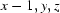; (iii) 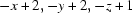. *Cg*1 and *Cg*2 are the centroids of theC32–C37 and C26–C31 benzene rings, respectively.

**Fig. 1.**
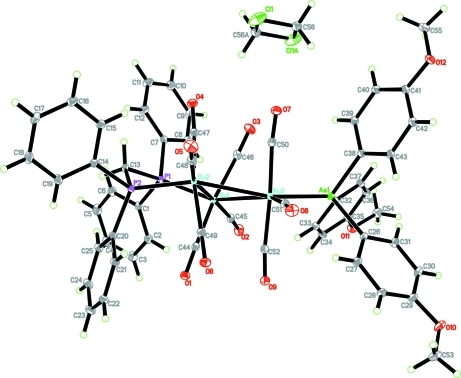
The structure of the title compound with 50% probability ellipsoids for non-H atoms. Atoms with suffix A are generated by the symmetry operation (2 - x, 1 - y, -z).

**Fig. 2.**
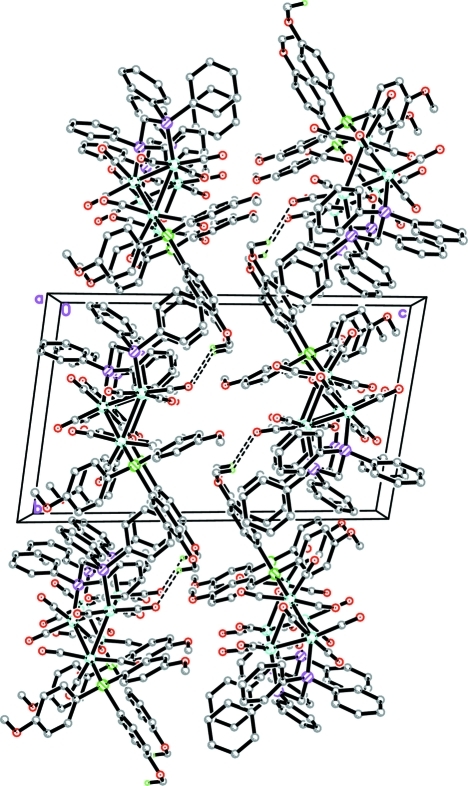
The crystal packing of the title compound, viewed down the a axis, showing the molecules linked along b axis. Hydrogen atoms that are not involved in the hydrogen-bonding (dashed lines) and solvent molecules have been omitted for clarity.

## supplementary crystallographic information

### Comment

*Triangulo*-triruthenium clusters are known for their interesting structural variations and related catalytic activity. A large number of substituted derivatives, Ru_3_(CO)_12-n_*L*_n_ (*L* = group 15 ligand) have been reported (Bruce *et al.*, 1985, 1988*a*,b). As part of our study of the substitution of transition metal-carbonyl clusters with mixed-ligand complexes, we have published several structures of *triangulo*-triruthenium-carbonyl clusters containing mixed P/As and P/Sb ligands (Shawkataly *et al.*, 1998, 2004, 2009). Herein we report the synthesis and structure of the title compound.

The asymmetric unit consists of one molecule of *triangulo*-triruthenium complex and half a molecule of dichloromethane solvent (Fig. 1). The dichloromethane solvent lies across a crystallographic inversion center (symmetry code: 2 - *x*, 1 - *y*, -*z*) leading to disorder of this solvent molecule over two positions. The bond lengths and angles of title compound are comparable to those found in related structures (Shawkataly *et al.*, 2009). The bis(diphenylphosphino)methane ligand bridges the Ru1—Ru2 bond and the monodentate arsine ligand bonds to the Ru3 atom. Both the arsine and phosphine ligands are equatorial with respect to the Ru_3_ triangle. Additionally, each Ru atom carries one equatorial and two axial terminal carbonyl ligands. The three arsine-substituted benzene rings make dihedral angles (C26–C31/C32–C37, C26–C31/C38–C43 and C32–C37/C38–C43) of 82.00 (6), 76.67 (7) and 66.09 (6)° with each other respectively. The dihedral angles between the two benzene rings (C1–C6/C7–C12 and C14–C19/C20–C25) are 80.12 (8) and 78.34 (7)° for the two diphenylphosphino groups respectively.

In the crystal packing (Fig. 2), the molecules are linked together into chains *via* intermolecular C53—H53C···O2 contacts along the *b* axis. An intermolecular C56—H56A···O7 hydrogen bond and weak intermolecular C—H···π interactions further stabilize the crystal structure (Table 1).

### Experimental

The reactions were conducted under an atmosphere of high purity nitrogen using standard Schlenk techniques and hexane-dried over sodium metal. Tris(4-methoxyphenyl)arsine (Blicke *et al.*, 1938) and µ-bis(diphenylphosphino)methanedecacarbonyltriruthenium(0) (Bruce *et al.*, 1983) were prepared by reported procedures. The title compound was obtained by refluxing equimolar quantities of Ru_3_(CO)_10_(µ-Ph_2_PCH_2_PPh_2_) (102.4 mg, 0.1 mmol) and tris(4-methoxyphenyl)arsine (39.63 mg, 0.1 mmol) in hexane under a nitrogen atmosphere. Crystals suitable for X-ray diffraction were grown by slow solvent / solvent diffusion of CH_3_OH into CH_2_Cl_2_.

### Refinement

All hydrogen atoms were positioned geometrically and refined using a riding model with C—H = 0.93–0.97 Å and *U*_iso_(H) = 1.2 or 1.5 *U*_eq_(C). A rotating group model was applied for the methyl groups.

### Crystal data

**Table:**

2[Ru_3_(C_21_H_21_AsO_3_)(C_25_H_22_P_2_)(CO)_9_]·CH_2_Cl_2_	*Z* = 1
*M**_r_* = 2756.85	*F*(000) = 1370
Triclinic, *P*1	*D*_x_ = 1.730 Mg m^−^^3^
Hall symbol: -P 1	Mo *K*α radiation, λ = 0.71073 Å
*a* = 10.7428 (1) Å	Cell parameters from 9697 reflections
*b* = 12.6731 (1) Å	θ = 2.8–35.1°
*c* = 20.6529 (2) Å	µ = 1.64 mm^−^^1^
α = 95.523 (1)°	*T* = 100 K
β = 101.315 (1)°	Block, purple
γ = 103.929 (1)°	0.30 × 0.23 × 0.14 mm
*V* = 2645.47 (4) Å^3^	

### Data collection

**Table:**

Bruker SMART APEXII CCD area-detector diffractometer	23203 independent reflections
Radiation source: fine-focus sealed tube	20247 reflections with *I* > 2σ(*I*)
graphite	*R*_int_ = 0.030
φ and ω scans	θ_max_ = 35.0°, θ_min_ = 1.8°
Absorption correction: multi-scan (*SADABS*; Bruker, 2005)	*h* = −17→17
*T*_min_ = 0.637, *T*_max_ = 0.797	*k* = −20→20
123575 measured reflections	*l* = −33→33

### Refinement

**Table:**

Refinement on *F*^2^	Primary atom site location: structure-invariant direct methods
Least-squares matrix: full	Secondary atom site location: difference Fourier map
*R*[*F*^2^ > 2σ(*F*^2^)] = 0.023	Hydrogen site location: inferred from neighbouring sites
*wR*(*F*^2^) = 0.055	H-atom parameters constrained
*S* = 1.02	*w* = 1/[σ^2^(*F*_o_^2^) + (0.0229*P*)^2^ + 1.6547*P*] where *P* = (*F*_o_^2^ + 2*F*_c_^2^)/3
23203 reflections	(Δ/σ)_max_ = 0.003
679 parameters	Δρ_max_ = 1.72 e Å^−^^3^
0 restraints	Δρ_min_ = −1.34 e Å^−^^3^

### Special details

**Table:**

Experimental. The crystal was placed in the cold stream of an Oxford Cyrosystems Cobra open-flow nitrogen cryostat (Cosier & Glazer, 1986) operating at 100.0 (1) K.
Geometry. All e.s.d.'s (except the e.s.d. in the dihedral angle between two l.s. planes) are estimated using the full covariance matrix. The cell e.s.d.'s are taken into account individually in the estimation of e.s.d.'s in distances, angles and torsion angles; correlations between e.s.d.'s in cell parameters are only used when they are defined by crystal symmetry. An approximate (isotropic) treatment of cell e.s.d.'s is used for estimating e.s.d.'s involving l.s. planes.
Refinement. Refinement of *F*^2^ against ALL reflections. The weighted *R*-factor *wR* and goodness of fit *S* are based on *F*^2^, conventional *R*-factors *R* are based on *F*, with *F* set to zero for negative *F*^2^. The threshold expression of *F*^2^ > σ(*F*^2^) is used only for calculating *R*-factors(gt) *etc*. and is not relevant to the choice of reflections for refinement. *R*-factors based on *F*^2^ are statistically about twice as large as those based on *F*, and *R*- factors based on ALL data will be even larger.

### Fractional atomic coordinates and isotropic or equivalent isotropic displacement parameters (Å^2^)

**Table:**

	*x*	*y*	*z*	*U*_iso_*/*U*_eq_	Occ. (<1)
Ru1	0.745666 (9)	0.425733 (8)	0.272810 (5)	0.01139 (2)	
Ru2	0.569657 (9)	0.487278 (8)	0.171185 (4)	0.01062 (2)	
Ru3	0.803319 (9)	0.646604 (8)	0.240008 (5)	0.01147 (2)	
As1	1.032151 (12)	0.748971 (10)	0.288763 (6)	0.01240 (2)	
P1	0.64526 (3)	0.23960 (3)	0.236633 (15)	0.01201 (5)	
P2	0.41178 (3)	0.32412 (3)	0.168781 (15)	0.01140 (5)	
O1	0.54704 (10)	0.46246 (9)	0.35615 (5)	0.01989 (18)	
O2	0.92704 (11)	0.39895 (10)	0.39911 (5)	0.0257 (2)	
O3	0.95575 (10)	0.42132 (10)	0.19116 (6)	0.0241 (2)	
O4	0.69735 (10)	0.36155 (9)	0.07987 (5)	0.02031 (18)	
O5	0.45036 (12)	0.58679 (10)	0.05376 (6)	0.0284 (2)	
O6	0.46711 (10)	0.63687 (9)	0.26185 (5)	0.0227 (2)	
O7	0.88918 (11)	0.59722 (9)	0.10904 (5)	0.0227 (2)	
O8	0.68903 (12)	0.83005 (9)	0.19351 (6)	0.0267 (2)	
O9	0.73330 (11)	0.68897 (9)	0.37569 (5)	0.0252 (2)	
O10	1.12273 (12)	1.16961 (9)	0.48187 (5)	0.0266 (2)	
O11	1.40724 (11)	0.61008 (10)	0.50258 (5)	0.0236 (2)	
O12	1.34237 (11)	0.92350 (9)	0.08579 (5)	0.0238 (2)	
C1	0.60776 (12)	0.14917 (10)	0.29888 (6)	0.0155 (2)	
C2	0.61938 (14)	0.19130 (12)	0.36533 (7)	0.0200 (2)	
H2A	0.6434	0.2670	0.3788	0.024*	
C3	0.59509 (17)	0.12028 (14)	0.41183 (8)	0.0268 (3)	
H3A	0.6027	0.1488	0.4561	0.032*	
C4	0.55954 (17)	0.00719 (14)	0.39210 (9)	0.0297 (3)	
H4A	0.5440	−0.0399	0.4232	0.036*	
C5	0.54714 (17)	−0.03567 (13)	0.32580 (9)	0.0282 (3)	
H5A	0.5230	−0.1114	0.3126	0.034*	
C6	0.57086 (15)	0.03478 (11)	0.27945 (8)	0.0220 (3)	
H6A	0.5622	0.0059	0.2352	0.026*	
C7	0.74231 (12)	0.16477 (10)	0.19620 (6)	0.0144 (2)	
C8	0.86801 (13)	0.16990 (12)	0.23303 (7)	0.0207 (2)	
H8A	0.8992	0.2122	0.2751	0.025*	
C9	0.94680 (14)	0.11242 (12)	0.20732 (8)	0.0245 (3)	
H9A	1.0302	0.1162	0.2323	0.029*	
C10	0.90165 (14)	0.04942 (12)	0.14450 (8)	0.0233 (3)	
H10A	0.9546	0.0110	0.1274	0.028*	
C11	0.77749 (15)	0.04389 (13)	0.10737 (8)	0.0246 (3)	
H11A	0.7473	0.0022	0.0651	0.030*	
C12	0.69770 (14)	0.10064 (12)	0.13319 (7)	0.0207 (2)	
H12A	0.6139	0.0958	0.1082	0.025*	
C13	0.48897 (12)	0.20925 (10)	0.17294 (6)	0.01391 (19)	
H13A	0.5057	0.1893	0.1295	0.017*	
H13B	0.4274	0.1462	0.1824	0.017*	
C14	0.28467 (12)	0.27167 (10)	0.09186 (6)	0.0140 (2)	
C15	0.32348 (13)	0.27282 (12)	0.03100 (6)	0.0189 (2)	
H15A	0.4117	0.3006	0.0307	0.023*	
C16	0.23111 (15)	0.23281 (13)	−0.02891 (7)	0.0229 (3)	
H16A	0.2579	0.2328	−0.0691	0.027*	
C17	0.09873 (15)	0.19283 (12)	−0.02904 (7)	0.0231 (3)	
H17A	0.0368	0.1672	−0.0692	0.028*	
C18	0.05952 (14)	0.19133 (13)	0.03066 (8)	0.0252 (3)	
H18A	−0.0290	0.1641	0.0305	0.030*	
C19	0.15155 (13)	0.23028 (12)	0.09126 (7)	0.0206 (2)	
H19A	0.1242	0.2287	0.1313	0.025*	
C20	0.32108 (12)	0.31633 (10)	0.23455 (6)	0.01380 (19)	
C21	0.25630 (12)	0.39752 (11)	0.24622 (6)	0.0165 (2)	
H21A	0.2544	0.4506	0.2182	0.020*	
C22	0.19488 (13)	0.39905 (12)	0.29952 (7)	0.0202 (2)	
H22A	0.1528	0.4536	0.3072	0.024*	
C23	0.19606 (14)	0.31958 (14)	0.34120 (7)	0.0230 (3)	
H23A	0.1559	0.3213	0.3771	0.028*	
C24	0.25741 (14)	0.23753 (13)	0.32909 (7)	0.0222 (3)	
H24A	0.2571	0.1836	0.3566	0.027*	
C25	0.31950 (12)	0.23542 (11)	0.27597 (6)	0.0170 (2)	
H25A	0.3601	0.1799	0.2681	0.020*	
C26	1.05575 (12)	0.88441 (10)	0.34835 (6)	0.0149 (2)	
C27	0.95099 (13)	0.92839 (11)	0.35364 (7)	0.0183 (2)	
H27A	0.8679	0.8939	0.3269	0.022*	
C28	0.96792 (14)	1.02354 (11)	0.39841 (7)	0.0201 (2)	
H28A	0.8968	1.0520	0.4015	0.024*	
C29	1.09228 (15)	1.07508 (11)	0.43817 (7)	0.0190 (2)	
C30	1.19835 (15)	1.03087 (11)	0.43365 (7)	0.0213 (2)	
H30A	1.2813	1.0647	0.4608	0.026*	
C31	1.18005 (13)	0.93698 (11)	0.38893 (7)	0.0189 (2)	
H31A	1.2511	0.9085	0.3858	0.023*	
C32	1.15368 (12)	0.68892 (10)	0.34692 (6)	0.0140 (2)	
C33	1.10840 (13)	0.63630 (11)	0.39765 (6)	0.0167 (2)	
H33A	1.0190	0.6188	0.3972	0.020*	
C34	1.19494 (14)	0.60997 (12)	0.44847 (7)	0.0191 (2)	
H34A	1.1638	0.5755	0.4821	0.023*	
C35	1.32949 (13)	0.63521 (11)	0.44929 (6)	0.0176 (2)	
C36	1.37556 (13)	0.68384 (11)	0.39775 (6)	0.0173 (2)	
H36A	1.4643	0.6981	0.3971	0.021*	
C37	1.28696 (13)	0.71086 (11)	0.34721 (7)	0.0166 (2)	
H37A	1.3177	0.7442	0.3131	0.020*	
C38	1.13298 (12)	0.80171 (10)	0.22457 (6)	0.0145 (2)	
C39	1.18482 (14)	0.73120 (10)	0.18772 (7)	0.0176 (2)	
H39A	1.1714	0.6582	0.1944	0.021*	
C40	1.25609 (14)	0.76895 (11)	0.14126 (7)	0.0195 (2)	
H40A	1.2924	0.7220	0.1181	0.023*	
C41	1.27291 (13)	0.87730 (11)	0.12957 (6)	0.0172 (2)	
C42	1.21673 (13)	0.94699 (11)	0.16395 (7)	0.0170 (2)	
H42A	1.2245	1.0184	0.1548	0.020*	
C43	1.14927 (12)	0.90978 (10)	0.21176 (6)	0.0158 (2)	
H43A	1.1146	0.9573	0.2355	0.019*	
C44	0.61781 (13)	0.45018 (10)	0.32303 (6)	0.0154 (2)	
C45	0.86226 (13)	0.41157 (11)	0.35056 (7)	0.0173 (2)	
C46	0.87634 (13)	0.42870 (11)	0.21981 (7)	0.0173 (2)	
C47	0.65515 (12)	0.41014 (10)	0.11587 (6)	0.0150 (2)	
C48	0.49368 (13)	0.54928 (11)	0.09827 (7)	0.0171 (2)	
C49	0.50943 (12)	0.57944 (11)	0.23128 (6)	0.0161 (2)	
C50	0.85260 (13)	0.61005 (10)	0.15679 (6)	0.0164 (2)	
C51	0.73810 (13)	0.76296 (11)	0.21037 (6)	0.0172 (2)	
C52	0.75643 (13)	0.66540 (11)	0.32505 (7)	0.0177 (2)	
C53	1.01782 (19)	1.21524 (13)	0.49108 (8)	0.0299 (3)	
H53A	1.0525	1.2839	0.5207	0.045*	
H53B	0.9577	1.1652	0.5100	0.045*	
H53C	0.9724	1.2275	0.4488	0.045*	
C54	1.54628 (15)	0.63830 (14)	0.50655 (8)	0.0258 (3)	
H54A	1.5902	0.6165	0.5461	0.039*	
H54B	1.5782	0.7163	0.5083	0.039*	
H54C	1.5636	0.6010	0.4679	0.039*	
C55	1.40600 (18)	0.85522 (14)	0.05196 (8)	0.0302 (3)	
H55A	1.4553	0.8970	0.0245	0.045*	
H55B	1.3408	0.7934	0.0244	0.045*	
H55C	1.4645	0.8296	0.0843	0.045*	
Cl1	0.88282 (6)	0.50832 (5)	−0.04776 (3)	0.04631 (12)	
C56	1.0352 (3)	0.5652 (2)	−0.00469 (16)	0.0247 (5)	0.50
H56A	1.0344	0.6309	0.0223	0.030*	0.50
H56B	1.0877	0.5875	−0.0361	0.030*	0.50

### Atomic displacement parameters (Å^2^)

**Table:**

	*U*^11^	*U*^22^	*U*^33^	*U*^12^	*U*^13^	*U*^23^
Ru1	0.01110 (4)	0.01066 (4)	0.01140 (4)	0.00242 (3)	0.00058 (3)	0.00214 (3)
Ru2	0.00987 (4)	0.01158 (4)	0.01061 (4)	0.00317 (3)	0.00215 (3)	0.00225 (3)
Ru3	0.01149 (4)	0.01044 (4)	0.01217 (4)	0.00238 (3)	0.00266 (3)	0.00171 (3)
As1	0.01191 (5)	0.01124 (5)	0.01406 (5)	0.00301 (4)	0.00338 (4)	0.00119 (4)
P1	0.01127 (12)	0.01126 (12)	0.01308 (12)	0.00303 (10)	0.00155 (10)	0.00214 (10)
P2	0.01008 (12)	0.01277 (12)	0.01112 (12)	0.00306 (10)	0.00179 (9)	0.00197 (10)
O1	0.0203 (4)	0.0230 (5)	0.0168 (4)	0.0054 (4)	0.0056 (3)	0.0029 (3)
O2	0.0234 (5)	0.0291 (5)	0.0211 (5)	0.0048 (4)	−0.0027 (4)	0.0092 (4)
O3	0.0196 (5)	0.0311 (5)	0.0276 (5)	0.0119 (4)	0.0094 (4)	0.0126 (4)
O4	0.0191 (4)	0.0240 (5)	0.0181 (4)	0.0076 (4)	0.0047 (4)	−0.0011 (4)
O5	0.0297 (6)	0.0364 (6)	0.0242 (5)	0.0152 (5)	0.0046 (4)	0.0154 (5)
O6	0.0209 (5)	0.0241 (5)	0.0250 (5)	0.0086 (4)	0.0085 (4)	−0.0008 (4)
O7	0.0276 (5)	0.0223 (5)	0.0198 (4)	0.0059 (4)	0.0102 (4)	0.0029 (4)
O8	0.0343 (6)	0.0234 (5)	0.0278 (5)	0.0160 (5)	0.0071 (5)	0.0086 (4)
O9	0.0283 (5)	0.0246 (5)	0.0196 (5)	−0.0005 (4)	0.0099 (4)	−0.0018 (4)
O10	0.0374 (6)	0.0177 (5)	0.0217 (5)	0.0076 (4)	0.0035 (4)	−0.0054 (4)
O11	0.0220 (5)	0.0315 (5)	0.0177 (4)	0.0100 (4)	0.0005 (4)	0.0071 (4)
O12	0.0273 (5)	0.0229 (5)	0.0234 (5)	0.0034 (4)	0.0135 (4)	0.0064 (4)
C1	0.0132 (5)	0.0152 (5)	0.0178 (5)	0.0030 (4)	0.0026 (4)	0.0054 (4)
C2	0.0221 (6)	0.0189 (6)	0.0187 (6)	0.0040 (5)	0.0042 (5)	0.0058 (4)
C3	0.0313 (8)	0.0284 (7)	0.0205 (6)	0.0045 (6)	0.0063 (6)	0.0104 (5)
C4	0.0332 (8)	0.0267 (7)	0.0303 (8)	0.0035 (6)	0.0096 (6)	0.0162 (6)
C5	0.0326 (8)	0.0175 (6)	0.0340 (8)	0.0021 (6)	0.0090 (6)	0.0101 (6)
C6	0.0247 (6)	0.0156 (5)	0.0249 (6)	0.0024 (5)	0.0065 (5)	0.0052 (5)
C7	0.0132 (5)	0.0124 (5)	0.0182 (5)	0.0040 (4)	0.0038 (4)	0.0028 (4)
C8	0.0153 (5)	0.0195 (6)	0.0256 (6)	0.0071 (5)	−0.0004 (5)	−0.0015 (5)
C9	0.0147 (5)	0.0211 (6)	0.0369 (8)	0.0082 (5)	0.0019 (5)	−0.0007 (5)
C10	0.0202 (6)	0.0187 (6)	0.0335 (7)	0.0085 (5)	0.0094 (5)	0.0016 (5)
C11	0.0250 (7)	0.0256 (7)	0.0243 (6)	0.0124 (6)	0.0044 (5)	−0.0024 (5)
C12	0.0195 (6)	0.0232 (6)	0.0199 (6)	0.0105 (5)	0.0017 (5)	−0.0011 (5)
C13	0.0128 (5)	0.0126 (5)	0.0150 (5)	0.0034 (4)	0.0008 (4)	0.0002 (4)
C14	0.0133 (5)	0.0142 (5)	0.0133 (5)	0.0029 (4)	0.0010 (4)	0.0018 (4)
C15	0.0168 (5)	0.0245 (6)	0.0146 (5)	0.0058 (5)	0.0021 (4)	0.0012 (4)
C16	0.0248 (6)	0.0285 (7)	0.0138 (5)	0.0082 (5)	0.0009 (5)	0.0001 (5)
C17	0.0224 (6)	0.0228 (6)	0.0179 (6)	0.0039 (5)	−0.0054 (5)	−0.0012 (5)
C18	0.0157 (6)	0.0306 (7)	0.0225 (6)	−0.0011 (5)	−0.0026 (5)	0.0030 (5)
C19	0.0137 (5)	0.0266 (6)	0.0180 (6)	0.0006 (5)	0.0012 (4)	0.0040 (5)
C20	0.0110 (4)	0.0169 (5)	0.0126 (5)	0.0025 (4)	0.0019 (4)	0.0025 (4)
C21	0.0145 (5)	0.0190 (5)	0.0170 (5)	0.0051 (4)	0.0047 (4)	0.0030 (4)
C22	0.0146 (5)	0.0260 (6)	0.0194 (6)	0.0050 (5)	0.0051 (4)	−0.0011 (5)
C23	0.0162 (6)	0.0363 (8)	0.0158 (5)	0.0043 (5)	0.0057 (4)	0.0035 (5)
C24	0.0182 (6)	0.0309 (7)	0.0177 (6)	0.0036 (5)	0.0053 (5)	0.0103 (5)
C25	0.0136 (5)	0.0202 (6)	0.0174 (5)	0.0037 (4)	0.0035 (4)	0.0061 (4)
C26	0.0159 (5)	0.0130 (5)	0.0155 (5)	0.0035 (4)	0.0037 (4)	0.0011 (4)
C27	0.0168 (5)	0.0173 (5)	0.0201 (6)	0.0046 (4)	0.0041 (4)	−0.0008 (4)
C28	0.0226 (6)	0.0169 (5)	0.0224 (6)	0.0065 (5)	0.0082 (5)	0.0004 (5)
C29	0.0279 (6)	0.0135 (5)	0.0154 (5)	0.0049 (5)	0.0061 (5)	0.0011 (4)
C30	0.0226 (6)	0.0159 (5)	0.0213 (6)	0.0035 (5)	−0.0004 (5)	−0.0009 (5)
C31	0.0178 (5)	0.0146 (5)	0.0223 (6)	0.0044 (4)	0.0015 (5)	0.0002 (4)
C32	0.0139 (5)	0.0133 (5)	0.0153 (5)	0.0041 (4)	0.0037 (4)	0.0020 (4)
C33	0.0163 (5)	0.0177 (5)	0.0163 (5)	0.0036 (4)	0.0050 (4)	0.0023 (4)
C34	0.0199 (6)	0.0227 (6)	0.0159 (5)	0.0050 (5)	0.0063 (4)	0.0050 (4)
C35	0.0188 (5)	0.0189 (6)	0.0144 (5)	0.0061 (4)	0.0017 (4)	0.0013 (4)
C36	0.0155 (5)	0.0195 (6)	0.0176 (5)	0.0061 (4)	0.0034 (4)	0.0033 (4)
C37	0.0157 (5)	0.0176 (5)	0.0185 (5)	0.0058 (4)	0.0059 (4)	0.0044 (4)
C38	0.0141 (5)	0.0135 (5)	0.0155 (5)	0.0028 (4)	0.0039 (4)	0.0024 (4)
C39	0.0220 (6)	0.0132 (5)	0.0190 (5)	0.0040 (4)	0.0081 (5)	0.0031 (4)
C40	0.0225 (6)	0.0172 (5)	0.0203 (6)	0.0049 (5)	0.0090 (5)	0.0020 (4)
C41	0.0159 (5)	0.0181 (5)	0.0160 (5)	0.0011 (4)	0.0040 (4)	0.0024 (4)
C42	0.0166 (5)	0.0150 (5)	0.0186 (5)	0.0023 (4)	0.0035 (4)	0.0043 (4)
C43	0.0148 (5)	0.0139 (5)	0.0190 (5)	0.0042 (4)	0.0043 (4)	0.0024 (4)
C44	0.0160 (5)	0.0139 (5)	0.0144 (5)	0.0028 (4)	0.0002 (4)	0.0023 (4)
C45	0.0164 (5)	0.0156 (5)	0.0187 (5)	0.0027 (4)	0.0021 (4)	0.0042 (4)
C46	0.0149 (5)	0.0178 (5)	0.0187 (5)	0.0042 (4)	0.0010 (4)	0.0063 (4)
C47	0.0119 (5)	0.0164 (5)	0.0156 (5)	0.0026 (4)	0.0017 (4)	0.0030 (4)
C48	0.0157 (5)	0.0187 (5)	0.0179 (5)	0.0055 (4)	0.0043 (4)	0.0044 (4)
C49	0.0148 (5)	0.0175 (5)	0.0160 (5)	0.0041 (4)	0.0034 (4)	0.0036 (4)
C50	0.0175 (5)	0.0132 (5)	0.0182 (5)	0.0037 (4)	0.0036 (4)	0.0023 (4)
C51	0.0181 (5)	0.0171 (5)	0.0166 (5)	0.0042 (4)	0.0052 (4)	0.0019 (4)
C52	0.0162 (5)	0.0166 (5)	0.0178 (5)	0.0004 (4)	0.0035 (4)	0.0017 (4)
C53	0.0457 (10)	0.0229 (7)	0.0245 (7)	0.0160 (7)	0.0098 (7)	−0.0016 (5)
C54	0.0207 (6)	0.0306 (7)	0.0235 (7)	0.0081 (6)	−0.0024 (5)	0.0035 (6)
C55	0.0376 (9)	0.0287 (7)	0.0267 (7)	0.0046 (6)	0.0191 (7)	0.0027 (6)
Cl1	0.0587 (3)	0.0458 (3)	0.0360 (2)	0.0284 (2)	0.0026 (2)	−0.00689 (19)
C56	0.0269 (14)	0.0214 (12)	0.0279 (14)	0.0061 (11)	0.0127 (11)	0.0010 (10)

### Geometric parameters (Å, °)

**Table:**

Ru1—C45	1.8852 (13)	C15—C16	1.3886 (19)
Ru1—C46	1.9379 (14)	C15—H15A	0.9300
Ru1—C44	1.9391 (13)	C16—C17	1.389 (2)
Ru1—P1	2.3250 (3)	C16—H16A	0.9300
Ru1—Ru2	2.83841 (13)	C17—C18	1.379 (2)
Ru1—Ru3	2.89355 (13)	C17—H17A	0.9300
Ru2—C48	1.8988 (13)	C18—C19	1.3958 (19)
Ru2—C49	1.9295 (13)	C18—H18A	0.9300
Ru2—C47	1.9322 (13)	C19—H19A	0.9300
Ru2—P2	2.3266 (3)	C20—C25	1.3963 (17)
Ru2—Ru3	2.82943 (14)	C20—C21	1.4014 (18)
Ru3—C51	1.8822 (13)	C21—C22	1.3909 (18)
Ru3—C52	1.9286 (13)	C21—H21A	0.9300
Ru3—C50	1.9417 (13)	C22—C23	1.387 (2)
Ru3—As1	2.44581 (16)	C22—H22A	0.9300
As1—C38	1.9399 (12)	C23—C24	1.387 (2)
As1—C32	1.9405 (12)	C23—H23A	0.9300
As1—C26	1.9458 (12)	C24—C25	1.3926 (19)
P1—C7	1.8300 (12)	C24—H24A	0.9300
P1—C1	1.8421 (13)	C25—H25A	0.9300
P1—C13	1.8499 (12)	C26—C27	1.3884 (18)
P2—C20	1.8175 (12)	C26—C31	1.3980 (18)
P2—C14	1.8272 (12)	C27—C28	1.3984 (18)
P2—C13	1.8416 (12)	C27—H27A	0.9300
O1—C44	1.1449 (16)	C28—C29	1.390 (2)
O2—C45	1.1515 (16)	C28—H28A	0.9300
O3—C46	1.1457 (17)	C29—C30	1.400 (2)
O4—C47	1.1473 (15)	C30—C31	1.3827 (19)
O5—C48	1.1446 (16)	C30—H30A	0.9300
O6—C49	1.1483 (16)	C31—H31A	0.9300
O7—C50	1.1427 (16)	C32—C37	1.3900 (17)
O8—C51	1.1502 (17)	C32—C33	1.3991 (17)
O9—C52	1.1500 (16)	C33—C34	1.3824 (19)
O10—C29	1.3580 (16)	C33—H33A	0.9300
O10—C53	1.420 (2)	C34—C35	1.3995 (19)
O11—C35	1.3584 (16)	C34—H34A	0.9300
O11—C54	1.4335 (19)	C35—C36	1.3943 (18)
O12—C41	1.3689 (16)	C36—C37	1.3958 (18)
O12—C55	1.4353 (19)	C36—H36A	0.9300
C1—C2	1.3940 (19)	C37—H37A	0.9300
C1—C6	1.4031 (19)	C38—C43	1.3960 (17)
C2—C3	1.397 (2)	C38—C39	1.4010 (17)
C2—H2A	0.9300	C39—C40	1.3914 (18)
C3—C4	1.389 (2)	C39—H39A	0.9300
C3—H3A	0.9300	C40—C41	1.3922 (19)
C4—C5	1.392 (2)	C40—H40A	0.9300
C4—H4A	0.9300	C41—C42	1.3973 (19)
C5—C6	1.389 (2)	C42—C43	1.3886 (18)
C5—H5A	0.9300	C42—H42A	0.9300
C6—H6A	0.9300	C43—H43A	0.9300
C7—C12	1.3956 (18)	C53—H53A	0.9600
C7—C8	1.3969 (18)	C53—H53B	0.9600
C8—C9	1.3884 (19)	C53—H53C	0.9600
C8—H8A	0.9300	C54—H54A	0.9600
C9—C10	1.387 (2)	C54—H54B	0.9600
C9—H9A	0.9300	C54—H54C	0.9600
C10—C11	1.384 (2)	C55—H55A	0.9600
C10—H10A	0.9300	C55—H55B	0.9600
C11—C12	1.3925 (19)	C55—H55C	0.9600
C11—H11A	0.9300	Cl1—C56	1.651 (3)
C12—H12A	0.9300	Cl1—C56^i^	1.741 (3)
C13—H13A	0.9700	C56—C56^i^	1.689 (6)
C13—H13B	0.9700	C56—Cl1^i^	1.741 (3)
C14—C19	1.3969 (18)	C56—H56A	0.9600
C14—C15	1.4000 (18)	C56—H56B	0.9602
			
C45—Ru1—C46	92.43 (6)	C18—C17—H17A	120.1
C45—Ru1—C44	91.81 (5)	C16—C17—H17A	120.1
C46—Ru1—C44	169.56 (5)	C17—C18—C19	120.60 (14)
C45—Ru1—P1	97.70 (4)	C17—C18—H18A	119.7
C46—Ru1—P1	93.18 (4)	C19—C18—H18A	119.7
C44—Ru1—P1	95.70 (4)	C18—C19—C14	120.04 (13)
C45—Ru1—Ru2	168.35 (4)	C18—C19—H19A	120.0
C46—Ru1—Ru2	93.26 (4)	C14—C19—H19A	120.0
C44—Ru1—Ru2	80.96 (4)	C25—C20—C21	118.98 (11)
P1—Ru1—Ru2	92.128 (8)	C25—C20—P2	122.11 (10)
C45—Ru1—Ru3	112.70 (4)	C21—C20—P2	118.83 (9)
C46—Ru1—Ru3	75.74 (4)	C22—C21—C20	120.29 (12)
C44—Ru1—Ru3	93.83 (4)	C22—C21—H21A	119.9
P1—Ru1—Ru3	147.777 (9)	C20—C21—H21A	119.9
Ru2—Ru1—Ru3	59.149 (3)	C23—C22—C21	120.30 (13)
C48—Ru2—C49	89.82 (5)	C23—C22—H22A	119.8
C48—Ru2—C47	92.03 (5)	C21—C22—H22A	119.8
C49—Ru2—C47	171.56 (5)	C24—C23—C22	119.75 (13)
C48—Ru2—P2	103.83 (4)	C24—C23—H23A	120.1
C49—Ru2—P2	97.23 (4)	C22—C23—H23A	120.1
C47—Ru2—P2	90.32 (4)	C23—C24—C25	120.39 (13)
C48—Ru2—Ru3	106.51 (4)	C23—C24—H24A	119.8
C49—Ru2—Ru3	76.49 (4)	C25—C24—H24A	119.8
C47—Ru2—Ru3	95.10 (4)	C24—C25—C20	120.25 (13)
P2—Ru2—Ru3	148.940 (9)	C24—C25—H25A	119.9
C48—Ru2—Ru1	164.95 (4)	C20—C25—H25A	119.9
C49—Ru2—Ru1	95.44 (4)	C27—C26—C31	118.76 (12)
C47—Ru2—Ru1	80.81 (4)	C27—C26—As1	121.69 (10)
P2—Ru2—Ru1	89.521 (8)	C31—C26—As1	119.50 (9)
Ru3—Ru2—Ru1	61.397 (3)	C26—C27—C28	121.30 (13)
C51—Ru3—C52	93.26 (6)	C26—C27—H27A	119.3
C51—Ru3—C50	93.52 (5)	C28—C27—H27A	119.3
C52—Ru3—C50	172.95 (5)	C29—C28—C27	119.21 (13)
C51—Ru3—As1	98.90 (4)	C29—C28—H28A	120.4
C52—Ru3—As1	91.63 (4)	C27—C28—H28A	120.4
C50—Ru3—As1	89.25 (4)	O10—C29—C28	125.03 (13)
C51—Ru3—Ru2	92.28 (4)	O10—C29—C30	115.01 (13)
C52—Ru3—Ru2	97.53 (4)	C28—C29—C30	119.95 (12)
C50—Ru3—Ru2	80.25 (4)	C31—C30—C29	120.11 (13)
As1—Ru3—Ru2	165.117 (5)	C31—C30—H30A	119.9
C51—Ru3—Ru1	147.69 (4)	C29—C30—H30A	119.9
C52—Ru3—Ru1	76.85 (4)	C30—C31—C26	120.66 (13)
C50—Ru3—Ru1	96.33 (4)	C30—C31—H31A	119.7
As1—Ru3—Ru1	111.900 (5)	C26—C31—H31A	119.7
Ru2—Ru3—Ru1	59.454 (3)	C37—C32—C33	118.73 (12)
C38—As1—C32	102.09 (5)	C37—C32—As1	123.00 (9)
C38—As1—C26	101.52 (5)	C33—C32—As1	117.45 (9)
C32—As1—C26	97.20 (5)	C34—C33—C32	120.79 (12)
C38—As1—Ru3	114.76 (4)	C34—C33—H33A	119.6
C32—As1—Ru3	122.65 (4)	C32—C33—H33A	119.6
C26—As1—Ru3	115.26 (4)	C33—C34—C35	119.99 (12)
C7—P1—C1	98.79 (6)	C33—C34—H34A	120.0
C7—P1—C13	102.47 (6)	C35—C34—H34A	120.0
C1—P1—C13	104.49 (6)	O11—C35—C36	124.43 (12)
C7—P1—Ru1	115.03 (4)	O11—C35—C34	115.63 (12)
C1—P1—Ru1	118.96 (4)	C36—C35—C34	119.94 (12)
C13—P1—Ru1	114.70 (4)	C35—C36—C37	119.25 (12)
C20—P2—C14	104.26 (6)	C35—C36—H36A	120.4
C20—P2—C13	105.29 (6)	C37—C36—H36A	120.4
C14—P2—C13	99.97 (6)	C32—C37—C36	121.23 (12)
C20—P2—Ru2	117.87 (4)	C32—C37—H37A	119.4
C14—P2—Ru2	117.41 (4)	C36—C37—H37A	119.4
C13—P2—Ru2	109.99 (4)	C43—C38—C39	118.67 (11)
C29—O10—C53	117.49 (13)	C43—C38—As1	120.24 (9)
C35—O11—C54	117.37 (11)	C39—C38—As1	121.00 (9)
C41—O12—C55	116.68 (12)	C40—C39—C38	120.91 (12)
C2—C1—C6	119.10 (12)	C40—C39—H39A	119.5
C2—C1—P1	121.73 (10)	C38—C39—H39A	119.5
C6—C1—P1	119.13 (10)	C39—C40—C41	119.75 (12)
C1—C2—C3	120.26 (13)	C39—C40—H40A	120.1
C1—C2—H2A	119.9	C41—C40—H40A	120.1
C3—C2—H2A	119.9	O12—C41—C40	124.72 (12)
C4—C3—C2	120.12 (15)	O12—C41—C42	115.49 (12)
C4—C3—H3A	119.9	C40—C41—C42	119.78 (12)
C2—C3—H3A	119.9	C43—C42—C41	120.12 (12)
C3—C4—C5	120.04 (14)	C43—C42—H42A	119.9
C3—C4—H4A	120.0	C41—C42—H42A	119.9
C5—C4—H4A	120.0	C42—C43—C38	120.67 (12)
C6—C5—C4	119.93 (14)	C42—C43—H43A	119.7
C6—C5—H5A	120.0	C38—C43—H43A	119.7
C4—C5—H5A	120.0	O1—C44—Ru1	175.51 (11)
C5—C6—C1	120.54 (14)	O2—C45—Ru1	175.86 (12)
C5—C6—H6A	119.7	O3—C46—Ru1	173.10 (12)
C1—C6—H6A	119.7	O4—C47—Ru2	175.10 (11)
C12—C7—C8	118.63 (12)	O5—C48—Ru2	178.46 (12)
C12—C7—P1	124.75 (10)	O6—C49—Ru2	173.63 (12)
C8—C7—P1	116.57 (10)	O7—C50—Ru3	174.12 (12)
C9—C8—C7	120.57 (13)	O8—C51—Ru3	174.56 (12)
C9—C8—H8A	119.7	O9—C52—Ru3	172.38 (12)
C7—C8—H8A	119.7	O10—C53—H53A	109.5
C10—C9—C8	120.28 (13)	O10—C53—H53B	109.5
C10—C9—H9A	119.9	H53A—C53—H53B	109.5
C8—C9—H9A	119.9	O10—C53—H53C	109.5
C11—C10—C9	119.77 (13)	H53A—C53—H53C	109.5
C11—C10—H10A	120.1	H53B—C53—H53C	109.5
C9—C10—H10A	120.1	O11—C54—H54A	109.5
C10—C11—C12	120.14 (14)	O11—C54—H54B	109.5
C10—C11—H11A	119.9	H54A—C54—H54B	109.5
C12—C11—H11A	119.9	O11—C54—H54C	109.5
C11—C12—C7	120.60 (13)	H54A—C54—H54C	109.5
C11—C12—H12A	119.7	H54B—C54—H54C	109.5
C7—C12—H12A	119.7	O12—C55—H55A	109.5
P2—C13—P1	114.19 (6)	O12—C55—H55B	109.5
P2—C13—H13A	108.7	H55A—C55—H55B	109.5
P1—C13—H13A	108.7	O12—C55—H55C	109.5
P2—C13—H13B	108.7	H55A—C55—H55C	109.5
P1—C13—H13B	108.7	H55B—C55—H55C	109.5
H13A—C13—H13B	107.6	C56—Cl1—C56^i^	59.66 (18)
C19—C14—C15	118.92 (12)	Cl1—C56—C56^i^	62.8 (2)
C19—C14—P2	122.99 (10)	Cl1—C56—Cl1^i^	120.34 (18)
C15—C14—P2	118.08 (9)	C56^i^—C56—Cl1^i^	57.53 (19)
C16—C15—C14	120.45 (13)	Cl1—C56—H56A	107.2
C16—C15—H15A	119.8	C56^i^—C56—H56A	126.2
C14—C15—H15A	119.8	Cl1^i^—C56—H56A	107.0
C15—C16—C17	120.21 (13)	Cl1—C56—H56B	107.3
C15—C16—H16A	119.9	C56^i^—C56—H56B	126.8
C17—C16—H16A	119.9	Cl1^i^—C56—H56B	107.4
C18—C17—C16	119.76 (13)	H56A—C56—H56B	107.0
			
C45—Ru1—Ru2—C48	86.4 (3)	C7—P1—C1—C2	134.94 (11)
C46—Ru1—Ru2—C48	−32.64 (16)	C13—P1—C1—C2	−119.66 (11)
C44—Ru1—Ru2—C48	138.62 (16)	Ru1—P1—C1—C2	9.85 (13)
P1—Ru1—Ru2—C48	−125.95 (16)	C7—P1—C1—C6	−42.85 (12)
Ru3—Ru1—Ru2—C48	38.61 (16)	C13—P1—C1—C6	62.56 (12)
C45—Ru1—Ru2—C49	−23.6 (2)	Ru1—P1—C1—C6	−167.94 (9)
C46—Ru1—Ru2—C49	−142.63 (6)	C6—C1—C2—C3	0.3 (2)
C44—Ru1—Ru2—C49	28.62 (5)	P1—C1—C2—C3	−177.51 (12)
P1—Ru1—Ru2—C49	124.06 (4)	C1—C2—C3—C4	0.2 (2)
Ru3—Ru1—Ru2—C49	−71.38 (4)	C2—C3—C4—C5	−0.4 (3)
C45—Ru1—Ru2—C47	148.8 (2)	C3—C4—C5—C6	0.2 (3)
C46—Ru1—Ru2—C47	29.75 (5)	C4—C5—C6—C1	0.2 (2)
C44—Ru1—Ru2—C47	−159.00 (5)	C2—C1—C6—C5	−0.5 (2)
P1—Ru1—Ru2—C47	−63.56 (4)	P1—C1—C6—C5	177.38 (12)
Ru3—Ru1—Ru2—C47	101.00 (4)	C1—P1—C7—C12	105.58 (12)
C45—Ru1—Ru2—P2	−120.8 (2)	C13—P1—C7—C12	−1.49 (13)
C46—Ru1—Ru2—P2	120.14 (4)	Ru1—P1—C7—C12	−126.62 (11)
C44—Ru1—Ru2—P2	−68.60 (4)	C1—P1—C7—C8	−71.82 (11)
P1—Ru1—Ru2—P2	26.836 (11)	C13—P1—C7—C8	−178.89 (10)
Ru3—Ru1—Ru2—P2	−168.606 (8)	Ru1—P1—C7—C8	55.97 (11)
C45—Ru1—Ru2—Ru3	47.8 (2)	C12—C7—C8—C9	0.1 (2)
C46—Ru1—Ru2—Ru3	−71.25 (4)	P1—C7—C8—C9	177.66 (12)
C44—Ru1—Ru2—Ru3	100.01 (4)	C7—C8—C9—C10	0.2 (2)
P1—Ru1—Ru2—Ru3	−164.558 (9)	C8—C9—C10—C11	0.0 (2)
C48—Ru2—Ru3—C51	26.29 (6)	C9—C10—C11—C12	−0.5 (2)
C49—Ru2—Ru3—C51	−59.44 (5)	C10—C11—C12—C7	0.9 (2)
C47—Ru2—Ru3—C51	119.93 (5)	C8—C7—C12—C11	−0.6 (2)
P2—Ru2—Ru3—C51	−140.93 (4)	P1—C7—C12—C11	−178.00 (12)
Ru1—Ru2—Ru3—C51	−163.44 (4)	C20—P2—C13—P1	−82.77 (8)
C48—Ru2—Ru3—C52	119.88 (6)	C14—P2—C13—P1	169.33 (7)
C49—Ru2—Ru3—C52	34.16 (6)	Ru2—P2—C13—P1	45.19 (7)
C47—Ru2—Ru3—C52	−146.48 (6)	C7—P1—C13—P2	−144.34 (7)
P2—Ru2—Ru3—C52	−47.34 (4)	C1—P1—C13—P2	113.03 (7)
Ru1—Ru2—Ru3—C52	−69.85 (4)	Ru1—P1—C13—P2	−18.98 (8)
C48—Ru2—Ru3—C50	−66.89 (6)	C20—P2—C14—C19	−3.77 (13)
C49—Ru2—Ru3—C50	−152.61 (5)	C13—P2—C14—C19	104.96 (12)
C47—Ru2—Ru3—C50	26.75 (5)	Ru2—P2—C14—C19	−136.22 (10)
P2—Ru2—Ru3—C50	125.89 (4)	C20—P2—C14—C15	176.58 (10)
Ru1—Ru2—Ru3—C50	103.38 (4)	C13—P2—C14—C15	−74.70 (11)
C48—Ru2—Ru3—As1	−112.57 (5)	Ru2—P2—C14—C15	44.12 (11)
C49—Ru2—Ru3—As1	161.71 (4)	C19—C14—C15—C16	−0.4 (2)
C47—Ru2—Ru3—As1	−18.93 (4)	P2—C14—C15—C16	179.29 (11)
P2—Ru2—Ru3—As1	80.21 (3)	C14—C15—C16—C17	1.0 (2)
Ru1—Ru2—Ru3—As1	57.70 (2)	C15—C16—C17—C18	−1.1 (2)
C48—Ru2—Ru3—Ru1	−170.27 (4)	C16—C17—C18—C19	0.4 (2)
C49—Ru2—Ru3—Ru1	104.01 (4)	C17—C18—C19—C14	0.3 (2)
C47—Ru2—Ru3—Ru1	−76.63 (4)	C15—C14—C19—C18	−0.3 (2)
P2—Ru2—Ru3—Ru1	22.512 (16)	P2—C14—C19—C18	−179.92 (12)
C45—Ru1—Ru3—C51	−138.48 (9)	C14—P2—C20—C25	104.89 (11)
C46—Ru1—Ru3—C51	134.90 (8)	C13—P2—C20—C25	0.13 (12)
C44—Ru1—Ru3—C51	−44.91 (8)	Ru2—P2—C20—C25	−122.92 (10)
P1—Ru1—Ru3—C51	62.12 (8)	C14—P2—C20—C21	−78.47 (11)
Ru2—Ru1—Ru3—C51	32.18 (7)	C13—P2—C20—C21	176.78 (10)
C45—Ru1—Ru3—C52	−63.56 (6)	Ru2—P2—C20—C21	53.72 (11)
C46—Ru1—Ru3—C52	−150.17 (6)	C25—C20—C21—C22	1.89 (19)
C44—Ru1—Ru3—C52	30.01 (5)	P2—C20—C21—C22	−174.86 (10)
P1—Ru1—Ru3—C52	137.04 (4)	C20—C21—C22—C23	−0.6 (2)
Ru2—Ru1—Ru3—C52	107.11 (4)	C21—C22—C23—C24	−0.8 (2)
C45—Ru1—Ru3—C50	114.61 (6)	C22—C23—C24—C25	1.0 (2)
C46—Ru1—Ru3—C50	27.99 (6)	C23—C24—C25—C20	0.3 (2)
C44—Ru1—Ru3—C50	−151.82 (5)	C21—C20—C25—C24	−1.72 (19)
P1—Ru1—Ru3—C50	−44.79 (4)	P2—C20—C25—C24	174.92 (10)
Ru2—Ru1—Ru3—C50	−74.73 (4)	C38—As1—C26—C27	114.32 (11)
C45—Ru1—Ru3—As1	22.87 (5)	C32—As1—C26—C27	−141.72 (11)
C46—Ru1—Ru3—As1	−63.75 (4)	Ru3—As1—C26—C27	−10.35 (12)
C44—Ru1—Ru3—As1	116.44 (4)	C38—As1—C26—C31	−68.20 (11)
P1—Ru1—Ru3—As1	−136.533 (16)	C32—As1—C26—C31	35.76 (11)
Ru2—Ru1—Ru3—As1	−166.468 (6)	Ru3—As1—C26—C31	167.14 (9)
C45—Ru1—Ru3—Ru2	−170.66 (4)	C31—C26—C27—C28	0.1 (2)
C46—Ru1—Ru3—Ru2	102.72 (4)	As1—C26—C27—C28	177.59 (10)
C44—Ru1—Ru3—Ru2	−77.09 (4)	C26—C27—C28—C29	0.2 (2)
P1—Ru1—Ru3—Ru2	29.935 (16)	C53—O10—C29—C28	4.6 (2)
C51—Ru3—As1—C38	−68.91 (6)	C53—O10—C29—C30	−176.87 (13)
C52—Ru3—As1—C38	−162.47 (6)	C27—C28—C29—O10	177.70 (13)
C50—Ru3—As1—C38	24.53 (5)	C27—C28—C29—C30	−0.8 (2)
Ru2—Ru3—As1—C38	69.37 (5)	O10—C29—C30—C31	−177.59 (13)
Ru1—Ru3—As1—C38	121.05 (4)	C28—C29—C30—C31	1.0 (2)
C51—Ru3—As1—C32	166.36 (6)	C29—C30—C31—C26	−0.7 (2)
C52—Ru3—As1—C32	72.81 (6)	C27—C26—C31—C30	0.2 (2)
C50—Ru3—As1—C32	−100.20 (6)	As1—C26—C31—C30	−177.39 (11)
Ru2—Ru3—As1—C32	−55.36 (5)	C38—As1—C32—C37	14.58 (12)
Ru1—Ru3—As1—C32	−3.67 (4)	C26—As1—C32—C37	−88.89 (11)
C51—Ru3—As1—C26	48.52 (6)	Ru3—As1—C32—C37	144.83 (9)
C52—Ru3—As1—C26	−45.03 (6)	C38—As1—C32—C33	−175.94 (10)
C50—Ru3—As1—C26	141.96 (6)	C26—As1—C32—C33	80.59 (10)
Ru2—Ru3—As1—C26	−173.19 (4)	Ru3—As1—C32—C33	−45.69 (11)
Ru1—Ru3—As1—C26	−121.51 (4)	C37—C32—C33—C34	2.24 (19)
C45—Ru1—P1—C7	−77.49 (6)	As1—C32—C33—C34	−167.71 (10)
C46—Ru1—P1—C7	15.39 (6)	C32—C33—C34—C35	−0.5 (2)
C44—Ru1—P1—C7	−170.10 (6)	C54—O11—C35—C36	1.7 (2)
Ru2—Ru1—P1—C7	108.77 (5)	C54—O11—C35—C34	−177.78 (13)
Ru3—Ru1—P1—C7	83.39 (5)	C33—C34—C35—O11	177.50 (12)
C45—Ru1—P1—C1	39.32 (6)	C33—C34—C35—C36	−2.0 (2)
C46—Ru1—P1—C1	132.20 (6)	O11—C35—C36—C37	−176.82 (13)
C44—Ru1—P1—C1	−53.29 (6)	C34—C35—C36—C37	2.6 (2)
Ru2—Ru1—P1—C1	−134.41 (5)	C33—C32—C37—C36	−1.58 (19)
Ru3—Ru1—P1—C1	−159.80 (5)	As1—C32—C37—C36	167.77 (10)
C45—Ru1—P1—C13	164.01 (6)	C35—C36—C37—C32	−0.8 (2)
C46—Ru1—P1—C13	−103.11 (6)	C32—As1—C38—C43	−127.57 (10)
C44—Ru1—P1—C13	71.40 (6)	C26—As1—C38—C43	−27.52 (11)
Ru2—Ru1—P1—C13	−9.72 (4)	Ru3—As1—C38—C43	97.48 (10)
Ru3—Ru1—P1—C13	−35.11 (5)	C32—As1—C38—C39	55.80 (11)
C48—Ru2—P2—C20	−109.00 (6)	C26—As1—C38—C39	155.86 (11)
C49—Ru2—P2—C20	−17.40 (6)	Ru3—As1—C38—C39	−79.15 (11)
C47—Ru2—P2—C20	158.83 (6)	C43—C38—C39—C40	2.7 (2)
Ru3—Ru2—P2—C20	58.38 (5)	As1—C38—C39—C40	179.41 (11)
Ru1—Ru2—P2—C20	78.02 (5)	C38—C39—C40—C41	−2.0 (2)
C48—Ru2—P2—C14	17.01 (6)	C55—O12—C41—C40	−2.6 (2)
C49—Ru2—P2—C14	108.61 (6)	C55—O12—C41—C42	177.35 (13)
C47—Ru2—P2—C14	−75.16 (6)	C39—C40—C41—O12	179.11 (13)
Ru3—Ru2—P2—C14	−175.61 (4)	C39—C40—C41—C42	−0.8 (2)
Ru1—Ru2—P2—C14	−155.96 (5)	O12—C41—C42—C43	−177.09 (12)
C48—Ru2—P2—C13	130.35 (6)	C40—C41—C42—C43	2.8 (2)
C49—Ru2—P2—C13	−138.05 (6)	C41—C42—C43—C38	−2.08 (19)
C47—Ru2—P2—C13	38.18 (6)	C39—C38—C43—C42	−0.68 (19)
Ru3—Ru2—P2—C13	−62.27 (5)	As1—C38—C43—C42	−177.38 (10)
Ru1—Ru2—P2—C13	−42.63 (4)	C56^i^—Cl1—C56—Cl1^i^	0.0

Symmetry codes: (i) −*x*+2, −*y*+1, −*z*.

### Hydrogen-bond geometry (Å, °)

**Table:**

Cg1 and Cg2 are the centroids of theC32–C37 and C26–C31 benzene rings, respectively.

**Table:**

*D*—H···*A*	*D*—H	H···*A*	*D*···*A*	*D*—H···*A*
C53—H53C···O2^ii^	0.96	2.60	3.346 (2)	135
C56—H56A···O7	0.96	2.60	3.116 (3)	114
C22—H22A···Cg1^iii^	0.93	2.91	3.5901 (16)	131
C53—H53B···Cg2^iv^	0.96	2.85	3.6951 (17)	147

Symmetry codes: (ii) *x*, *y*+1, *z*; (iii) *x*−1, *y*, *z*; (iv) −*x*+2, −*y*+2, −*z*+1.

## References

[bb1] Blicke, F. F. & Cataline, E. L. (1938). *J. Am. Chem. Soc.*, **60**, 419–422.

[bb2] Bruce, M. I., Liddell, M. J., Hughes, C. A., Patrick, J. M., Skelton, B. W. & White, A. H. (1988*a*). *J. Organomet. Chem.* **347**, 181–205.

[bb3] Bruce, M. I., Liddell, M. J., Shawkataly, O. bin, Hughes, C. A., Skelton, B. W. & White, A. H. (1988*b*). *J. Organomet. Chem.* **347**, 207–235.

[bb4] Bruce, M. I., Matisons, J. G. & Nicholson, B. K. (1983). *J. Organomet. Chem.* **247**, 321–343.

[bb5] Bruce, M. I., Shawkataly, O. bin & Williams, M. L. (1985). *J. Organomet. Chem.* **287**, 127–131.

[bb6] Bruker (2005). *APEX2*, *SAINT* and *SADABS* Bruker AXS Inc., Madison, Wisconsin, USA.

[bb7] Cosier, J. & Glazer, A. M. (1986). *J. Appl. Cryst.* **19**, 105–107.

[bb8] Shawkataly, O. bin, Khan, I. A., Yeap, C. S. & Fun, H.-K. (2009). *Acta Cryst.* E**65**, m1620–m1621. 10.1107/S1600536809047229 PMC2971801 21578643

[bb9] Shawkataly, O. bin, Ramalingam, K., Fun, H.-K., Abdul Rahman, A., & Razak, I. A. (2004). *J. Cluster Sci.* **15**, 387–394.

[bb10] Shawkataly, O. bin., Ramalingam, K., Lee, S. T., Parameswary, M., Fun, H.-K. & Sivakumar, K. (1998). *Polyhedron*, **17**, 1211–1216.

[bb11] Sheldrick, G. M. (2008). *Acta Cryst.* A**64**, 112–122. 10.1107/S0108767307043930 18156677

[bb12] Spek, A. L. (2009). *Acta Cryst.* D**65**, 148–155. 10.1107/S090744490804362X PMC2631630 19171970

